# Biopsy of bone tumors: a literature review

**DOI:** 10.1590/1806-9282.2024S131

**Published:** 2024-06-07

**Authors:** Alex Guedes, Suely Akiko Nakagawa

**Affiliations:** 1 Hospital Santa Izabel, Santa Casa de Misericórdia da Bahia, Orthopedic Oncology Group – Salvador (BA), Brazil.; 2 Reference Center for Bone Tumors and Sarcomas, A.C.Camargo Cancer Center – São Paulo (SP), Brazil.

## INTRODUCTION

In the differential diagnosis of bone tumors, the following parameters are relevant: age, onset and duration of symptoms, lesion location (affected bone), affected part of the bone, radiographic aspect, and tumor growth pattern.

Clinical evaluation and conventional radiographic examination, together, allow correct diagnosis in >80% of patients. The investigation should proceed where: (a) clinical and imaging findings suggest biological aggressiveness; (b) findings are normal/indeterminate despite clinical presentation; or (c) it is necessary to restage the tumor. Additional imaging modalities are required, seeking accurate information on tumor tissue composition, its anatomical relationships, and metabolic and functional profiles, in addition to the presence of distant dissemination—bone biopsy constitutes the final stage of evaluation, completing the tumor staging^1,2^.

The aim of bone biopsy is to obtain a representative tumor tissue sample that enables histopathological, immunohistochemical, cytogenetic, and molecular processing, defining the diagnosis and histological grading. An inadequate technique can make correct tissue analysis unfeasible, hinder definitive surgery, and increase local recurrence and metastase rates among other complications, in addition to potentially making it impossible to preserve the affected limb and/or reduce the patient's chances of survival. The biopsy should be performed by an experienced surgeon, preferably the one who will perform the definitive procedure^3–6^.

More recently, we have observed the development of liquid biopsy, which, among other applications, has been used in the diagnosis and follow-up of bone and soft tissue sarcomas^7–9^.

This paper updates the reader on the biopsy techniques currently employed in the diagnosis and graduation of bone tumors.

### Planning of bone biopsies

A proper bone biopsy requires meticulous planning. The shortest distance to the lesion is not necessarily the ideal path for sample acquisition. Whichever technique is listed, it must follow fundamental principles for its execution^10^ (Table 1).

**Table 1:** Fundamental principles of bone biopsies.

Principle 1	All biopsies should be located so that they can be resected en bloc with the operative specimen at the time of definitive surgery.	Performed along the access route planned for the definitive surgery; In the extremities, incisions should be made in the longitudinal direction, following the longest axis of the segments addressed. In other sites, use accesses that avoid contamination of more than one compartment and facilitate oncological resection of tumors. If the use of a drain is necessary, its exit hole should be located along and close to the skin incision (~ 1 cm).
Principle 2	Avoid contamination of compartments not involved by the tumor.	Avoid: Violation of compartmental barriers; Anatomical dissection; Hematoma; Traverse soft tissue structures necessary for reconstruction.
Principle 3	The material obtained through biopsy should provide a diagnosis.	Ensure that sufficient tissue is obtained; Ensure that a representative sample of the tumor (periphery of the lesion) is obtained; When considering the demand for differential diagnosis with osteoarticular infection, it is recommended to obtain samples for culture and antibiogram.
Principle 4	Avoid iatrogenic complications such as stress fractures or infection at the biopsy site.	The cortical orifice should be small, oval, or circular; Adequate asepsis and antisepsis; Preoperative antibiotic prophylaxis.
Principle 5	Rigorous hemostasis should be achieved prior to wound closure.	Identify history of bleeding disorders and the use of anticoagulants, among other conditions in the preoperative period; Occlude the cortical orifice after the procedure (polymethylmethacrylate, bone wax, or hemostatic sponge); If there is a demand for the use of a tourniquet, remove it before performing definitive hemostasis; If necessary, use a drain along and near the skin incision (~1 cm).

Source: adapted from Campanacci^10^.

Bone biopsy should be postponed until the imaging evaluation has been completed in order to (a) allow accurate collection planning, seeking the most representative area of the lesion, in line with definitive surgical access; (b) facilitate differential diagnosis, allowing histopathological correlation; and (c) avoid previous manipulation that generates edema and image artifacts^1,2^.

It is critical to obtain a sufficient and representative tumor sample. Benign aggressive or malignant primary bone tumors are usually heterogeneous—multiple samples need to be collected to establish a diagnosis. Bone metastases from carcinoma and multiple myeloma, in contrast, are homogeneous, and it is usually sufficient to collect a single tissue sample or aspirate for diagnostic definition^11^.

Samples should be collected from the peripheral area, which usually contains viable tumors. It is important to identify and avoid reactive areas and necrotic or hemorrhagic components.

**Figure 1:**
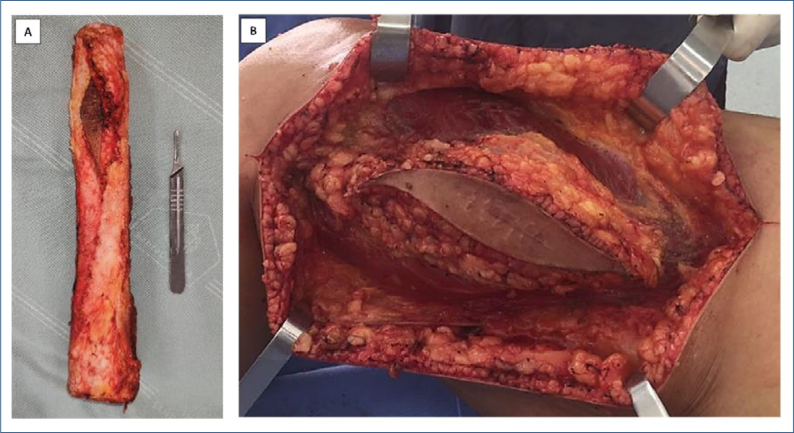
(A) Adamantinoma of the tibia: the piece was resected en bloc with the scar and biopsy path. (B) Osteosarcoma of the distal femur; intraoperative aspect of the procedure of wide tumor resection, biological reconstruction, and fixation - three-dimensional resection of the biopsy pathway, resected together with the specimen in sequence.

Until proven otherwise, all bone lesions that require bone biopsy should be considered malignant—biopsy routes should always be considered contaminated, requiring complete subsequent removal, en bloc with the resected specimen, in definitive surgery (Figure 1). Anatomical dissection should be averted, as well as violating not affected compartments, intercompartmental planes, neurovascular bundles, and joints. Crossing soft tissue structures necessary for limb reconstruction should also be avoided. Therefore, it is important to plan the biopsy site along the planned access route for definitive surgery. The incision should follow the main axis of the segment being approached^3–6,11^.

Perforation of the affected bone during biopsy can lead to iatrogenic fracture. This possibility should be minimized by making small oval or circular bone holes.

In addition to preoperative antibiotic prophylaxis, infections at the biopsy site should be prevented by adequate asepsis and antisepsis.

It is very important to establish absolute hemostasis to avoid hematoma, because of local dissemination risk, particularly in open biopsies—any hematoma around a tumor should be considered contaminated; large hematomas can dissect compartments, affecting the entire extremity and making it impossible to preserve the limb. It is of paramount importance to identify a history of bleeding disorders and the use of anticoagulants, among other conditions, before the biopsy is scheduled. If there is a need to use a tourniquet, venous emptying using an Esmarch band is contraindicated, due to the risk of proximal tumor dissemination through lymphatic and venous routes—gravitational emptying should be chosen. The tourniquet should be removed before wound closure, allowing adequate hemostasis. Drains generally are not used—in the rare cases where they are needed, their exit holes should be along and near (1 cm) the incision—the drainage path is considered contaminated and should be excised together with the surgical specimen, in the same way as the biopsy path. Bleeding through the bone orifice can be contained by occlusion with bone wax, hemostatic sponge, or polymethylmethacrylate^3–6,11^.

### Bone biopsy techniques

Diagnostic accuracy should be the most important parameter in defining the bone biopsy technique. There are two types: (a) percutaneous/closed biopsies, minimally invasive procedures, guided or not by images and (b) open biopsies, in which samples are obtained through bloody approach, incisional (lesion sample collection) or excisional (complete lesion resection).

The proximity of adjacent critical structures, the lesion topography, and comfort for the patient should influence the selection of the collection site. In the cases where multiple lesions are present, the most accessible or safest lesion should be chosen for biopsy accomplishment.

### Closed or percutaneous bone biopsies

Image-guided percutaneous bone biopsy has become the preferred diagnostic method for a bone neoplasm. It is a minimally invasive procedure with a high level of diagnostic accuracy and a low rate of complications.

Planning a percutaneous biopsy often requires more time and effort than performing this procedure itself. Decisions regarding the selection of guiding modality, needle type, path, specific target in the lesion, and expected pathological findings should be defined before the procedure^12^.

Fluoroscopy, computed tomography (CT), magnetic resonance imaging (MRI), single photon emission CT (SPECT), SPECT/CT, and positron emission tomography–CT (PET/CT) enable precise orientation of percutaneous bone biopsies. The quality and availability of imaging modalities vary between practices—logistical details may limit your choice^13–23^.

**Figure 2:**
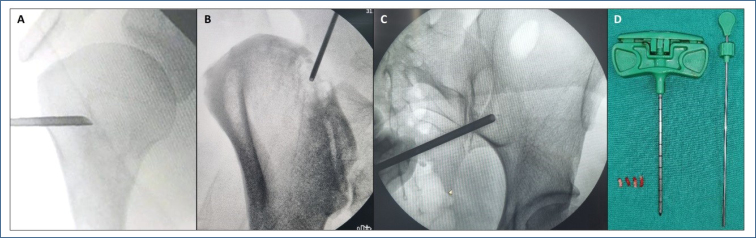
(A-D). Core needle biopsies. (A) A 40-year-old female patient with suspected enchondroma/chondrosarcoma in the proximal segment of the right humerus. A radioscopy-guided biopsy of the affected segment was performed with trephine. Diagnosis of grade 1 chondrosarcoma was confirmed. (B) A 52-year-old male patient with suspected metastatic lesion of undetermined origin in the right iliac. A radioscopy-guided biopsy with trephine was performed. Diagnosis of mesenchymal chondrosarcoma was made. (C) A 30-year-old female patient with a bone lesion in the left iliac. A radioscopy guided biopsy with trephine was performed. A diagnosis of simple bone cyst was made. (D) Jamshidi needle for bone biopsy mounted next to the extraction probe ("pusher"); chuck and trocar hinge at the T-handle; The trocar has a tapered stylet tip and the chuck features a "triple-crown" type cannula tip - these features provide a sharp and effective cutting tip for improved cortical penetration and medullary advancement that requires 25% less physical force. Appearance of the collection of four bone tumor samples.

Fluoroscopy (Figure 2) and CT are the preferred modalities for guided biopsies. In hard-to-reach sites (spine and waist belts), CT guidance, in addition to increasing diagnostic accuracy, reduces the rate of complications.

The use of MRI, SPECT, SPECT/CT, and PET/CT in staging has increased the diagnosis of occult bone lesions, defining anatomical landmarks that help to perform CT-guided biopsies, when not guided by these modalities per se.

Although MRI is devoid of ionizing radiation and provides superior characterization and delineation of lesions, MRI-guided biopsy is generally not feasible (equipment compatibility, patient positioning, cost, and execution time), or necessary.

Radionuclide-guided bone biopsy is highly accurate, achieving sensitivity and specificity of up to 100%. The use of a gamma probe is useful in differential diagnosis, especially to confirm or rule out metastatic disease, especially when it is not possible to define an appropriate site for biopsy using other imaging methodologies, in addition to providing limited exposure to ionizing radiation.

### Fine-needle aspiration biopsy

In this modality, a thin, hollow needle is inserted directly into the lesion, obtaining a sample for cytological examination by aspirating^24^.

Fine-needle aspiration biopsy obtains greater precision in homogeneous lesions (carcinoma metastases and multiple myeloma) and can be used in local recurrences or distant dissemination, in which cytological findings can be compared with those previously obtained.

It is a relatively atraumatic, minimally invasive outpatient procedure, with low cost and morbidity and lower contamination risk.

Its main limitation is that it does not allow the evaluation of tissue architecture, making it difficult or impossible to perform ancillary studies. The incidence of false-negative results is high, and even when results are positive, the diagnosis may not be accurate^3–6,24^.

### Core needle biopsy

A large needle is inserted through a small incision in the site planned for biopsy, preferably guided by imaging modalities^13–23^. Multiple samples are obtained, in different directions, through a single bone hole, reducing the risk of iatrogenic fracture.

This technique is useful in lesions in which a small sample is sufficient to confirm the diagnosis. The tissue architecture is preserved, allowing histological diagnosis, tumor grading, and ancillary analyses.

Recent studies suggest that the diagnostic yield of CNB is like that of incisional biopsy, reaching 70–98%. Yield can be maximized by collecting at least three samples; specimens >10 mm are 6.3 times more likely to allow a diagnosis than specimens <5 mm. Lesions ≥3 cm have a diagnostic yield five times higher than lesions <3 cm. Needles with a gauge of less than 18 mm may result in lower diagnostic yield.

The advantages of CNB are as follows: (a) minimally invasive, outpatient procedure; (b) lower cost; (c) low risk of route contamination; (c) tumor samples collection in places that are difficult to locate more safely and accurately, when guided by imaging tests; and (d) lower risk for complications than incisional biopsies (0–10% vs. 16%)^13–23,25^.

### Open bone biopsies

The open approach allows the collection of samples in greater quantity and of better quality, facilitating the pathologist's evaluation. However, it has greater potential for local contamination and systemic dissemination, as well as for other complications, such as hematoma, fracture, and infection^3–6^.

### Incisional bone biopsy

Incisional biopsy is indicated: (a) in the most difficult cases, where there is diagnostic uncertainty; (b) where accurate histological study requires a larger sample for diagnosis; or (c) when previously performed biopsy did not define the diagnosis.

This modality is still considered the "gold standard" for the diagnosis of musculoskeletal tumors because a diagnostic yield of 91–96% can be achieved.

Incisional biopsy can be performed in association with a frozen section, ensuring that a representative tumor sample has been obtained. It should be performed through wide access, along the incision line planned for the definitive treatment. It is mandatory to use the smallest incision compatible with obtaining an adequate sample, preferably in the affected compartment topography and, as far as possible, distal—especially in cases where amputation is envisaged. Transverse incisions are contraindicated because they require wider soft tissue resection at the time of definitive surgery. Following the same principle, more than one access should be avoided^3–6^.

The disadvantages associated with this procedure include greater potential for contamination and a higher rate of local tumor recurrence, as well as complications related to the surgical wound (16%). In addition, it may require an increase in the extent of definitive resection, compromising the function of the affected limb^3–6^.

### Excisional bone biopsy

Excisional biopsy is a special form of open biopsy. Depending on the lesion location and size, marginal, or even wide, resection is achieved.

Excisional biopsies are indicated when the clinical/imaging characteristics (small tumors, with an unequivocally benign appearance and biological behavior) and the lesion topography (e.g., out of neurovascular bundle path) to be biopsied allow its wide resection in a single time and safely.

### Liquid biopsy

Liquid biopsy consists of the collection of blood (most used) samples or other body fluids from which circulating tumor cells, cell-free nucleic acids, exosomes, and metabolites are extracted for analysis of any genomic, molecular, and metabolomic alterations. The collection is minimally invasive and circumvents many of the limitations of conventional biopsy. It can be performed at any time during cancer therapy, allowing dynamic monitoring of molecular changes in the tumor rather than relying on a static point in time^7–9^.

This technique can provide a more accurate representation of the overall cancer genome than a single tissue biopsy. Longitudinal screening of genetic and epigenetic alterations through liquid biopsies has multiple applications. Liquid biopsy can better assess molecular heterogeneity, identify acquired tumor mutations, and characterize primary and recurrent tumors; monitor recurrence and metastases; predict treatment response; identify genetic determinants for targeted therapies; clarify the mechanisms of resistant tumor evolution in real time under treatment pressure; and screen asymptomatic individuals for early detection of cancer^7–9^.

Liquid biopsy has been introduced into the routine diagnosis and follow-up of patients with bone and soft tissue sarcomas, establishing itself as a promising tool in the management of these neoplasms.

## CONCLUSION

The domain of knowledge about bone biopsy planning is essential to obtain representative tissue samples, favoring tumor diagnosis and grading, as well as ancillary studies accomplishment (immunohistochemical, cytogenetic, and molecular processing), which allows defining the most adequate therapeutic protocol for each case while avoiding unnecessary complications related to vices in procedure execution.

Liquid biopsy is a promising tool in the management of bone sarcomas, providing a more accurate representation of the overall cancer genome, with several therapeutic implications.
